# The association between patent foramen ovale and unexplained syncope in pediatric patients

**DOI:** 10.1186/s13052-023-01572-y

**Published:** 2024-01-07

**Authors:** Runmei Zou, Shuo Wang, Ping Liu, Donghai Chen, Jun Yan, Hong Cai, Yuwen Wang, Cheng Wang

**Affiliations:** 1 Department of Pediatric Cardiovasology, Children’s Medical Center, The Second Xiangya Hospital, Central South University, No.139 Renmin Middle Road, Changsha, Hunan 410011 China; 2 Department of Pediatrics, Xiangya Hospital, Central South University, Changsha, Hunan 410008 China; 3 Department of Pediatrics, The First Affiliated Hospital, Hunan University of Medicine, Huaihua, Hunan 418000 China

**Keywords:** Patent foramen ovale, Unexplained syncope, Pediatric patients, Echocardiography

## Abstract

**Background:**

Patent foramen ovale (PFO) is associated with transient ischemia attack (TIA) or stroke, paradoxical embolism, and migraines. PFO closure decreases the recurrent incidence of cerebral ischemic events and reduces the incidence of syncope in adults. However, whether PFO is associated with syncope in pediatric patients has not been investigated.

**Methods:**

1001 pediatric patients (aged 4 to 17 years, mean age 10.31 ± 2.61 years, 519 males) who complained of unexplained syncope, palpitation, headache, dizziness and chest pain and were hospitalized in the Syncope Ward, The Second Xiangya Hospital, Central South University between January 2013 and April 2022 were recruited. Children with definite etiology of syncope, neurological, cardiogenic, psychological and other system diseases were excluded. PFO was measured by transthoracic echocardiography and right-heart contrast echocardiography was performed to identify the presence of right-to-left shunting. The demographic data and medical records were retrospectively reviewed and analyzed.

**Results:**

276 cases were included in the simple syncope group, 379 cases in the headache/dizziness group, 265 cases in the chest pain group, and 81 cases in the palpitation group. The incidence of PFO between the four groups was insignificant (4.71%, 4.74%, 4.15%, 6.17%, respectively, *P* = 0.903). Multivariate Logistic regression demonstrated that PFO is not associated with the increased risk of syncope (*P* = 0.081).

**Conclusion:**

PFO may not increase the risk of syncope in pediatric patients. Further study may include a large and multicenter sample to investigate the association between PFO and unexplained syncope.

## Background

Syncope is frequent in children and accounts for 1% of emergencies [1]. Though most patients have benign causes of syncope, the recurrent onset of syncope may affect children’s life quality and bring syncope-associated injury [2]. It is important to identify the risk factors of syncope for effective preventive measures to reduce syncopal attacks.

Foramen ovale is an intracardiac structure for fetal circulation. Most foramina ovalia functionally close within a few days to several months after birth due to pulmonary circulatory blood flow and left atrial pressure increase. Patent foramen ovale (PFO) is a remnant of fetal foramen ovale with an overall incidence of 27.3% [3]. PFO is associated with transient ischemia attack (TIA) or stroke, paradoxical embolism, and migraine in adults [4], and PFO closure may reduce the risk of PFO-associated stroke in patients younger than 60 years with an embolic-appearing stroke [5, 6]. Besides, PFO closure also decreases the incidences of platypnea-orthodeoxia, fainting episodes, syncope, and migraine headaches [7]. PFO is more common in children, and the incidence is 34.3% during the first three decades of life [3]. Whether PFO is associated with syncope in pediatric patients has not been investigated yet. In this study, we aim to investigate the association between PFO and syncope in children.

## Study population and methods

### Study population and data collection

**Fig. 1:**
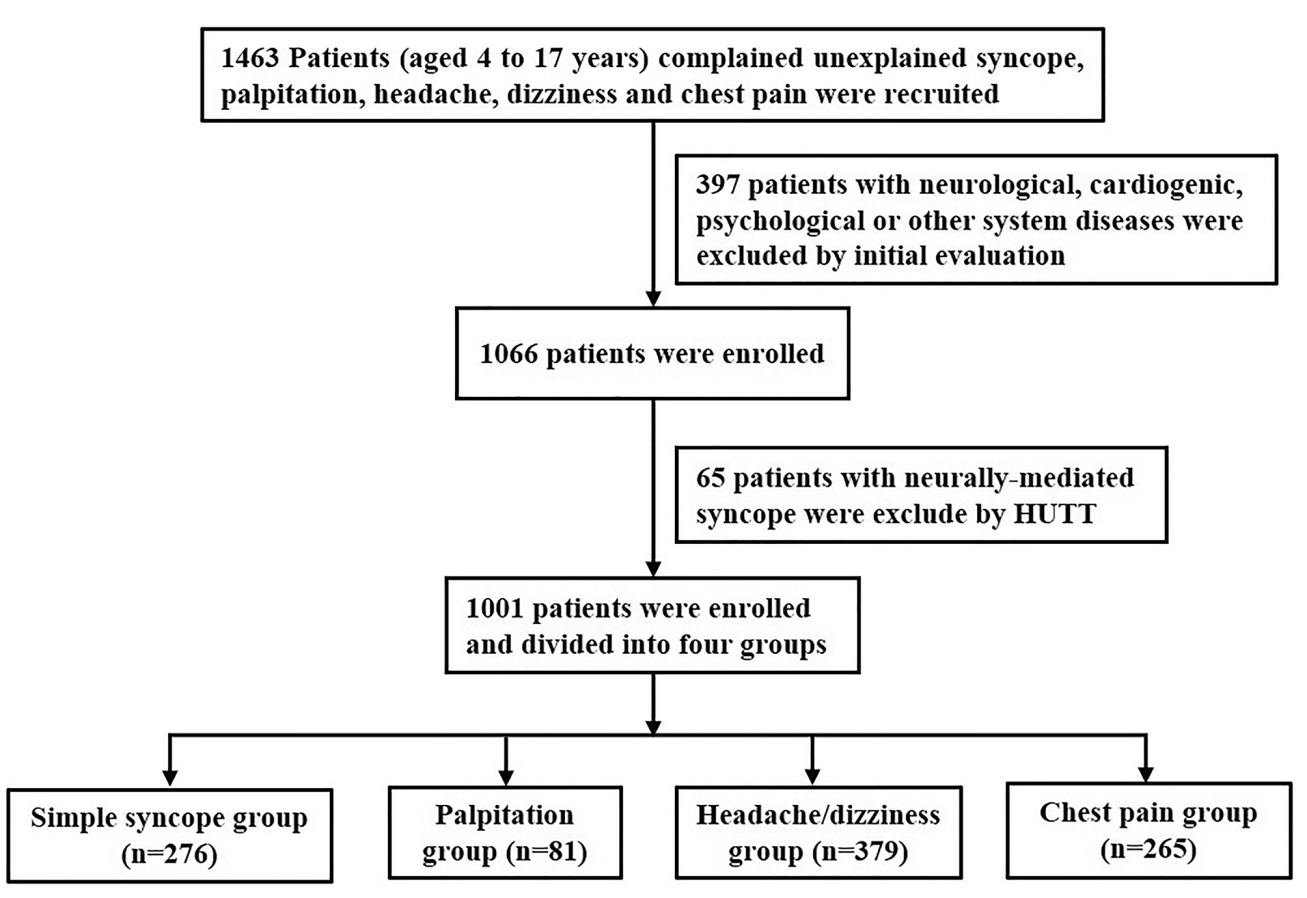
Flow chart of the study population exclusion and inclusion. (Note: HUTT, head-up tilt test)

Patients who complained of unexplained syncope, palpitation, headache/dizziness, and chest pain were hospitalized in the Syncope Ward, The Second Xiangya Hospital, Central South University between January 2013 and April 2022. The demographic data and medical records were retrospectively reviewed by two researchers. Neurological, cardiogenic, psychological, and other system diseases were excluded after an initial evaluation consisting of history, physical examination, baseline laboratory testing, electrocardiogram (ECG), Holter ECG, echocardiography, chest X-ray, electroencephalogram, and cranial CT or MRI. Head-up tilt test was performed according to the previous study [8] to exclude neurally-mediated syncope. Patients were divided into four groups according to their symptoms: simple syncope group, headache/dizziness group, chest pain group and palpitation group. The exclusion and inclusion process are shown as Fig. 1.

### Patent foramen ovale measurement

PFO was monitored by transthoracic echocardiography, and the standard echocardiographic protocol was performed according to the American Society of Echocardiography [9]. Right-heart contrast echocardiography was performed to identify the presence of right-to-left shunting, by using agitated saline (80%) combined with air (10%) and the patient’s own blood (10%) when the patient was at rest or performing a Valsalva maneuver. Microbubbles were detected in the left atrium during 3 to 5 cardiac cycles [10, 11].

### Statistical analysis

Statistical analysis was performed by SPSS 24.0 (IBM Corp., Armonk, NY, United States). Continuous variables for data following normal distribution were described as mean ± SD and analyzed by one-way ANOVA with polynomial contrasts and post hoc LSD among groups. Categorical data were described by frequencies and percentages and analyzed using *x*^2^ test or Fisher’s exact test. Univariate analysis and multiple Logistic regression were utilized to analyze the association between unexplained syncope and related factors. *P*-value < 0.05 was considered to be a statistically significant difference.

## Results

A total of 1001 patients, aged 4 to 17 years (mean age 10.31 ± 2.61 years) with 519 males (51.85%), were recruited in the study. As shown in Tables 1, 276 cases [mean age 10.60 ± 2.75 years, 138 males (50.00%)] were included in the simple syncope group, 379 cases [mean age 10.25 ± 2.38 years, 200 males (52.77%)] in the headache/dizziness group, 265 cases [mean age 10.32 ± 2.60 years, 143 males (53.96%)] in the chest pain group, and 81 cases [mean age 9.61 ± 3.08 years, 38 males (46.91%)] in the palpitation group. Patients in the palpitation group were younger than those patients in the simple syncope group, headache/dizziness group, and palpitation group (all *P* < 0.05). However, there were no significant differences in sex ratio, height, body weight, systolic blood pressure, diastolic blood pressure, heart rate, ejection fraction, and fractional shortening between the four groups (all *P* > 0.05). 13 (4.71%) patients in the simple syncope group, 18 (4.74%) patients in the headache/dizziness group, 11 (4.15%) children in the chest pain group, and 5 (6.17%) children in the palpitation group have PFO. There was no significant difference in the proportion of PFO between these groups (*P* = 0.903). 4 patients in the headache/dizziness group presented a positive bubble study, but no patient in the simple syncope group had a positive bubble study.

**Table 1:** The demographic data and clinical characteristics in children with unexplained syncope, headache/dizziness, chest pain and palpitation

Variables	Simple syncope group	Headache/dizziness group	Chest pain group	Palpitation group	*P*-value
Case, n	276	379	265	81	
Sex					0.621
Male, n (%)	138 (50.00)	200(52.77)	143(53.96)	38 (46.91)	
Female, n (%)	138 (50.00)	179(47.23)	122(46.04)	43 (53.08)	
Age, years	10.60 ± 2.75^*^	10.25 ± 2.38^*^	10.32 ± 2.60^*^	9.61 ± 3.08	0.027
Height, cm	145.74 ± 18.20	143.03 ± 17.29	148.10 ± 14.57	148.43 ± 11.45	0.713
Body weight, kg	38.07 ± 12.68	34.51 ± 12.31	41.65 ± 17.74	34.78 ± 9.17	0.305
SBP, mmHg	112.43 ± 11.82	111.06 ± 10.71	108.00 ± 8.75	108.57 ± 8.73	0.476
DBP, mmHg	65.83 ± 11.14	65.39 ± 7.39	64.75 ± 9.72	64.57 ± 10.15	0.976
HR, beats/min	73.88 ± 13.22	78.61 ± 12.58	74.05 ± 11.05	86.57 ± 31.20	0.138
PFO, n (%)	13 (4.71)	18 (4.74)	11(4.15)	5(6.17)	0.903
EF, %	68.08 ± 4.07	66.15 ± 3.53	65.57 ± 3.31	70.50 ± 3.53	0.231
FS, %	38.25 ± 2.92	39.38 ± 12.23	35.14 ± 2.96	40.00 ± 2.82	0.711

SBP, systolic blood pressure; DBP, diastolic blood pressure; HR, heart rate;

PFO, patent foramen ovale; EF, ejection fraction; FS, fractional shortening

*Compared with the palpitation group, *P*<0.05

Univariate analysis for unexplained syncope was performed. As shown in Table 2, we found a positive association between age and unexplained syncope. With one-year increase in age, the risk of syncope was raised by 6.0% (OR = 1.060, 95%CI: 1.004–1.119, *P* = 0.036). PFO was not associated with unexplained syncope in children (*P* = 0.989). Multivariate Logistic regression demonstrated that PFO was not the independent factor of syncope (*P* = 0.081) (Table 3). These results suggested that the incidence of syncope is not associated with PFO in children.

**Table 2:** Univariate analysis for unexplained syncope in children

Variables	Statistics	OR (95%CI)	*P*-value
Sex			
Male, n (%)	519(51.85)	1.0	
Female, n (%)	482(48.15)	0.903 (0.684–1.191)	0.470
Age, years	10.31 ± 2.61	1.060 (1.004–1.119)	0.036
Height, cm	145.55 ± 16.63	1.001(0.976–1.027)	0.930
Body weight, kg	37.41 ± 13.67	1.006 (0.975–1.037)	0.715
SBP, mmHg	110.73 ± 10.63	1.025 (0.984–1.066)	0.234
DBP, mmHg	65.35 ± 9.49	1.008 (0.965–1.054)	0.708
HR, beats/min	76.48 ± 14.78	0.978 (0.946–1.012)	0.204
FPO			
No, n (%)	954(95.30)	1	
Yes, n (%)	47(4.70)	1.005(0.522–1.933)	0.989

SBP, systolic blood pressure; DBP, diastolic blood pressure; HR, heart rate; PFO, patent foramen ovale

**Table 3:** Multivariate Logistic regression analysis between unexplained syncope and related factors in children

Variables	OR (95% CI)	*P*-value
Sex (Male/Female)	1.685 (0.677–4.194)	0.262
Age, years	1.095 (0.778–1.541)	0.603
Height, cm	0.961 (0.889–1.037)	0.309
Body weight, kg	1.027 (0.960–1.099)	0.436
PFO (No/Yes)	2.542 (0.890–7.256)	0.081

PFO, patent foramen ovale

## Discussion

In this study, we assess the relationship between syncope and PFO in pediatric patients with unexplained syncope, headache, dizziness, palpitation, and chest pain. The results demonstrated that the incidence of PFO had no significant differences in children with or without syncope. Unlike adults, PFO did not increase the risk of syncope in pediatric patients.

Previous studies suggested that PFO is associated with stroke in adults. 40–50% of patients who underwent a cryptogenic stroke have PFO [12]. Half of the patients with a history of PFO-related stroke also had symptoms of syncope and palpitation [13]. A study demonstrated that the prevalence of PFO in patients with explained syncope was 75.4% [14], while it was 20–25% in the general population [15]. Based on the above results, it is concluded that PFO is highly correlated with unexplained syncope in adults, especially during exercise or an increase in abdominal pressure.

The main mechanism of PFO-related syncope may be associated with paradoxical embolism. First, when laughing, coughing, or doing other activities, the pressure of the right atrium increases transiently, which will push open the primary septum to the left atrium and cause right-to-left shunting. Vasoactive substances and thrombi from the venous system enter the intracranial arterial system through right-to-left shunting, which results in arterial spasms and transient cerebral ischemia, inducing syncope [16]. Second, the emboli caused by the repeated opening of the foramen ovale or lower limb veins fall off and enter the left atrium. Then the emboli are pushed into the systemic circulation from the left atrium, leading to arterial embolism, which may lead to stroke, myocardial infarction, and syncope [17, 18]. Third, venous blood shunted from the right atrium to the left heart system makes the cerebral blood supply become mixed arteriovenous blood supplies, causing transient cerebral ischemia and hypoxia, resulting in syncope [19].

The results of our study demonstrated that PFO was not associated with syncope in pediatric patients, which contradicts the results in adults. In our study, 4 patients in the headache/dizziness group presented a positive bubble study, suggesting the presence of right-to-left shunting. However, the 4 patients did not experience syncope. It is believed that the thrombi, air thrombi, and vasoactive substances of the venous system in children are fewer compared with adults. Fewer emboli from the venous system enter systemic circulation through PFO though the existence of right-to-left shunting. Besides, the diameter of PFO is clinically significant and positively related to the severity of diseases. When the diameter is less than 4 mm, the blood diversion is slight, and the probability of paradoxical embolism is lower [10]. On the other hand, no patient in the simple syncope group presented a positive bubble study, suggesting no existence of right-to-left shunting in these patients. The results indicated that syncope is unrelated to PFO in these children.

There are several limitations in the study. First, this was a retrospective and single-center study, which resulted in bias and underestimation of some important factors. Patients included in the study complained of unexplained syncope, palpitation, headache, dizziness, and chest pain, but healthy individuals with PFO were not included. The incidence of PFO in our study was lower than that of the general population. Besides, the sample size of patients with PFO and the proportion of patients with positive bubble studies was small. We believed that because it is difficult for children to perform the Valsalva maneuver correctly.

## Conclusion

PFO may not increase the risk of syncope in pediatric patients. Further study may include a large and multicenter sample to investigate the association between PFO and unexplained syncope.

## Data Availability

The original contributions presented in the study are included in the article/supplementary material, further inquiries can be directed to the corresponding author.

## Abbreviations

PFO: Patent foramen ovale
ECG: Electrocardiogram
